# A transient α-helical molecular recognition element in the disordered N-terminus of the Sgs1 helicase is critical for chromosome stability and binding of Top3/Rmi1

**DOI:** 10.1093/nar/gkt817

**Published:** 2013-09-14

**Authors:** Jessica A. Kennedy, Gary W. Daughdrill, Kristina H. Schmidt

**Affiliations:** ^1^Department of Cell Biology, Microbiology and Molecular Biology, University of South Florida, Tampa, FL 33620, USA, ^2^Center for Drug Discovery and Innovation, University of South Florida, Tampa, FL 33612, USA and ^3^Cancer Biology and Evolution Program, H. Lee Moffitt Cancer Center and Research Institute, Tampa, FL 33612, USA

## Abstract

The RecQ-like DNA helicase family is essential for the maintenance of genome stability in all organisms. Sgs1, a member of this family in *Saccharomyces cerevisiae*, regulates early and late steps of double-strand break repair by homologous recombination. Using nuclear magnetic resonance spectroscopy, we show that the N-terminal 125 residues of Sgs1 are disordered and contain a transient α-helix that extends from residue 25 to 38. Based on the residue-specific knowledge of transient secondary structure, we designed proline mutations to disrupt this α-helix and observed hypersensitivity to DNA damaging agents and increased frequency of genome rearrangements. *In vitro* binding assays show that the defects of the proline mutants are the result of impaired binding of Top3 and Rmi1 to Sgs1. Extending mutagenesis N-terminally revealed a second functionally critical region that spans residues 9–17. Depending on the position of the proline substitution in the helix functional impairment of Sgs1 function varied, gradually increasing from the C- to the N-terminus. The multiscale approach we used to interrogate structure/function relationships in the long disordered N-terminal segment of Sgs1 allowed us to precisely define a functionally critical region and should be generally applicable to other disordered proteins.

## INTRODUCTION

The maintenance of genome stability is essential for organismal survival. A complex and diverse system of proteins has evolved to accomplish this function. Sgs1 of *Saccharomyces cerevisiae* is a 3–5′ DNA helicase that belongs to the evolutionarily conserved RecQ helicase family whose members function in the maintenance of genome stability. Named after the RecQ helicase of *Escherichia coli*, members of this helicase family have been identified in all organisms, including five homologs in humans (RecQ1, BLM, WRN, RecQL4, RecQL5) (1). Mutations in BLM, WRN and RecQL4 are associated with Bloom syndrome, Werner syndrome and Rothmund–Thompson syndrome, respectively, which are characterized by elevated levels of aberrant recombination events, chromosome instability and extraordinary predisposition to cancer development early in life (1).

*Saccharomyces cerevisiae* cells that lack Sgs1 exhibit several phenotypes that are similar to those of cells from persons with Bloom syndrome, most notably dysregulated homologous recombination, hypersensitivity to DNA-damaging agents, meiotic defects and cell cycle delay (2,3). These defects are caused when the helicase activity of Sgs1 is inactivated by mutations in the ATPase domain or the RecQ C-terminal domain, which together make up the helicase core. Also located in the C-terminal half of Sgs1 is the Helicase and RNAase D C-terminal (HRDC) domain thought to be involved in DNA substrate binding and protein–protein interactions. These domains are conserved in most RecQ homologs; they are structurally ordered and crystal structures of this region have been reported for *E. coli* RecQ and human RecQ1 (4,5). In contrast, the N-terminal half of Sgs1 is devoid of conserved catalytic domains and provides binding sites for proteins with roles in DNA metabolism, including the topoisomerases Top2 and Top3, replication protein Rpa70, Rad16 and Srs2 (2,6–8). Interaction with the Top3 homologs has also been shown for human BLM, RecQ1 and RecQ5, and the RecQ homolog of *Schizosaccharomyces pombe*, Rqh1 (9–12). Superhelical relaxation activity and Holliday-junction dissolution activity of these topoisomerase/helicase complexes is greatly enhanced by interaction with the RecQ-mediated genome instability 1 (Rmi1) protein (13–15).

One of the most important functions of the Sgs1 N-terminus is the interaction with the Top3/Rmi1 complex (BLM/Topo IIIα/Rmi1/Rmi2 in humans, Rqh1/Top3/Rmi1 in *S. pombe*) (13–16). The Top3 binding site is within the first 100–158 residues of Sgs1 (17–19). The loss of this region produces more severe phenotypes that exhibit slower growth and higher sensitivity to DNA damage than those produced by loss of Sgs1 alone (3). This may be due to toxic intermediates produced by Sgs1 that accumulate during homologous recombination and require Top3 decatenation for resolution. Despite the fact that Sgs1 and BLM bind Top3 and its human homolog Topo IIIα, respectively, there is little primary sequence similarity between the N-terminal regions where these interactions are predicted to occur. Both N-termini are predicted to be intrinsically disordered (20), which may help explain their level of sequence divergence (21,22). Such intrinsically disordered proteins/regions (IDPs/IDRs) are widespread in eukaryotes and function arises from an ensemble of conformations that contain varying degrees of secondary structure and rarely form transient tertiary contacts (21,23–28). A high percentage of eukaryotic proteins are predicted to contain significant stretches (>30 residues) of disorder; in *S. cerevisiae*, 50–60% of the total proteome are IDPs/IDRs, and a survey of cancer-associated human proteins found that ∼79% of the proteins in the database are IDPs/IDRs (29,30).

Using multidimensional heteronuclear nuclear magnetic resonance (NMR) spectroscopy, we have identified a short segment within the first 125 residues of the intrinsically disordered N-terminus of unbound Sgs1 that has transient α-helical structure whose integrity is essential for Sgs1 function *in vivo*. We have rationally designed single amino acid substitutions that disrupt transient α-helices. Some of these mutations eliminate Top3 binding to Sgs1, cause DNA damage hypersensitivity and induce spontaneous chromosomal rearrangements.

## MATERIALS AND METHODS

### Expression and purification of peptides for NMR spectroscopy

Methods were based on a previously described procedure for the expression of an IDP (31). Plasmid pKHS443, expressing Sgs1^1^^−125^, was constructed by inserting the first 375 bp of *SGS1* into pET28a (Novagen) using *Nde*I and *Bam*HI sites. Plasmid pKHS463, expressing Sgs1^1^^−80^, was constructed by introducing a stop codon after 240 bp in pKHS443. pKHS443 or pKHS463 was transformed into *E. coli* BL21 (DE) cells and grown at 37°C in 2 l of M9 media (42 mM Na_2_HPO_4_, 22 mM KH_2_PO_4_, 8 mM NaCl, 2 mM MgSO_4_, 11 mM d-glucose, 0.1 mM CaCl_2_, 10 µM FeCl_3_, 1 mg of Vitamin B1/L, pH 7.3) plus 200 mg of ampicillin, supplemented with N^15^ ammonium chloride and C^13^ glucose. Protein expression was induced at OD_600_ = 0.6 for 3 h with 1 mM Isopropyl-beta-D-thiogalactopyranoside (IPTG) at 37°C. Cells were harvested via centrifugation at 8000 rpm before being resuspended in buffer A1 (50 mM NaH_2_PO_4_, 300 mM NaCl, 10 mM imidazole, pH 8.0) and lysed at 19 000 psi via French press. The lysate was cleared via centrifugation (18 000 rpm, 1 h, 4°C) and the supernatant was loaded onto a 30 ml Ni-NTA column on an AKTA FPLC. The column was washed with 5 column volumes of buffer A2 (50 mM NaH_2_PO_4_, 300 mM NaCl, 20 mM imidazole, pH 8.0), and the peptide was eluted in buffer B (50 mM NaH_2_PO_4_, 300 mM NaCl, 300 mM imidazole, pH 8.0). Fractions containing the eluted protein were pooled and dialyzed into 50 mM Tris (pH 8.0) and 100 mM NaCl. The fractions were treated with 1 ml CleanCleave thrombin beads (Sigma) at room temperature for 8 h to remove the N-terminal (HIS)_6_ tag. Cleaved proteins were dialyzed into gel filtration buffer (50 mM NaH_2_PO_4_, 300 mM NaCl, 1 mM EDTA, 0.02% NaN_3_, 4 mM DL-Dithiothreitol (DTT), pH 7), then concentrated to a volume of 10 ml and loaded onto a 120-ml GE Hiload 16/60 Superdex 70 column via fast protein liquid chromatography (FPLC) and harvested over four 2.5-ml runs. Fractions containing the peptide were pooled and dialyzed into NMR buffer (50 mM NaH_2_PO_4_, 100 mM NaCl, 1 mM EDTA, 0.02% NaN_3_, 4 mM DTT, pH 6.8) before being concentrated to 600 µl (150 µM for Sgs1^1^^−125^; 690 µM for Sgs1^1^^−80^, 160 µM for Sgs1^1^^−80^-F30P).

### NMR analysis

NMR data for Sgs1^1^^−80^ and Sgs1^1^^−80^-F30P were collected at 25°C on a Varian VNMRS 800 MHz spectrometer equipped with a triple resonance pulse field *Z*-axis gradient cold probe. To make the amide ^1^H and ^15^N as well as ^13^C, ^13^C_β_ and ^13^CO resonance assignments, sensitivity enhanced ^1^H-^15^N heteronuclear single quantum correlation (HSQC) and three-dimensional HNCACB and HNCO experiments were performed on a uniformly ^15^N- and ^13^C-labeled sample of Sgs1^1^^−80^ at 470 µM (or Sgs1^1^^−80^-F30P at 160 µM) in 90% H_2_O/10% D2O, phosphate buffered saline (PBS) buffer, at a pH of 6.8 (32–34). For the HNCACB experiment, data were acquired in ^1^H, ^13^C and ^15^N dimensions using 9615.3846 (*t*_3_) × 16 086.4648 (*t*_2_) × 2000 (*t*_1_) Hz sweep widths, and 512 (*t*_3_) × 128 (*t*_2_) × 32 (*t*_1_) complex data points. For the HNCO, the sweep widths were 9615.3846 (*t*_3_) × 2000 (*t*_2_) × 2000 (*t*_1_) Hz, complex data points were identical to the HNCACB. The sweep widths and complex data points of the HSQC were 9615.3846 (*t*_2_) × 2100 (*t*_1_) Hz and 1024 (*t*_2_) × 128 (*t*_1_), respectively. Processing and analysis of the HNCACB data resulted in 66 nonproline amide ^1^H, ^15^N, ^13^Cα and ^13^Cb resonance assignments plus 8 proline ^13^Cα and ^13^Cβ resonance assignments. ^1^H-^15^N steady-state nulcear Overhauser effect (NOE) experiments were recorded at 25°C on a Varian VNMRS 600 MHz spectrometer equipped with a triple resonance pulse field *Z*-axis gradient cold probe in the presence and absence of a 120 off-resonance ^1^H saturation pulse every 5 ms for 3 s. A total of 512 (*t*_2_) × 128 (*t*_1_) complex points were recorded with 128 scans per increment with the sweep widths set to 7225.4335 (*t*_2_) × 1700 (*t*_1_) Hz. The ^1^H-^15^N heteronuclear Overhauser effect (NHNOE) values were determined by taking the quotient of the intensity for resolved resonances in the presence and absence of proton saturation. Three measurements were made on each protein and the values were averaged. Resonance assignments for Sgs1^1^^−125^ were carried out at 25°C on a Varian VNMRS 600 MHz spectrometer equipped with a triple resonance pulse field *Z*-axis gradient cold probe. To make the amide ^1^H and ^15^N as well as ^13^C_α_, ^13^C_β_ and ^13^CO resonance assignments, sensitivity-enhanced ^1^H-^15^N HSQC and three-dimensional HNCACB and HNCO experiments were performed on a uniformly ^15^N- and ^13^C-labeled sample at 150 µM in 90% H_2_O/10% D_2_O, PBS buffer, at pH 6.8. For the HNCACB experiment, data were acquired in ^1^H, ^13^C and ^15^N dimensions using 7225.4335 (*t*_3_) × 12 064.1295 (*t*_2_) × 1499.9813 (*t*_1_) Hz sweep widths, and 512 (*t*_3_) × 108 (*t*_2_) × 32 (*t*_1_) complex data points. For the HNCO, the sweep widths were 7225.4335 (*t*_3_) × 1500 (*t*_2_) × 1499.9813 (*t*_1_) Hz, and 512 (*t*_3_) × 74 (*t*_2_) × 32 (*t*_1_) complex data points. For the HNCACO, the sweep widths were 7225.4335 (*t*_3_) × 12 000 (*t*_2_) × 1499.9813 (*t*_1_) Hz, and 512 (*t*_3_) × 70 (*t*_2_) × 28 (*t*_1_) complex data points. Processing and analysis of the data resulted in 87 nonproline amide ^1^H, ^15^N, ^13^C_α_ and ^13^Cb resonance assignments plus 12 proline ^13^Cα and ^13^Cβ resonance assignments. All NMR spectra were processed with nmrPipe and analyzed using nmrView software (31,35,36). Apodization was achieved in the ^1^H, ^13^C and ^15^N dimensions using a squared sine bell function shifted by 70°. Apodization was followed by zero filling to twice the number of real data points and linear prediction was used in the ^15^N dimension of the HNCACB.

### Hydroxyurea hypersensitivity assay

Yeast strain KHSY1338 (*ura3-52*, *leu2Δ1*, *trp1Δ63*, *his3Δ200*, *lys2ΔBgl*, *hom3-10*, *ade2Δ1*, *ade8*, *YEL069C::URA3*, *sgs1::HIS3*) was transformed with derivatives of plasmid pRS415-SGS1 (Supplementary Table S1) by standard lithium-acetate transformation (37) and selected on synthetic complete media lacking leucine (SC-Leu). Transformants were grown in liquid SC-Leu to OD_600_ = 0.5, then plated in 10-fold dilutions on YPD (yeast extract/peptone/dextrose) and on YPD supplemented with 100 mM hydroxyurea (HU). Colony growth at 30°C was documented after 3–5 days.

### Top3 and Rmi1 binding assay

Plasmid pKHS462, expressing GST-Sgs1^1^^−250^, was constructed by inserting the first 750 bp of *SGS1* into pGEX-6p-2 (GE Healthcare) using *Bam*HI and *Xho*I sites. The Sgs1 fragment was expressed in *E. coli* BL21 (DE) cells in LB media (10 g/l tryptone, 5 g/l NaCl, 5 g/l Yeast extract) supplemented with 1.5 mg ampicillin for 3 h in the presence of 1 mM IPTG. The cell pellet was resuspended in 100 µl GST buffer (125 mM Tris, 150 mM NaCl, pH 8.0) plus HALT protease inhibitors (Pierce) and sonicated for 10 × 3 pulses. Lysate was cleared by centrifugation at 14 000 rpm for 10 min at 4°C. Glutathione magnetic beads (Pierce) were then incubated with 625 µg of cleared lysate for 1 h at 4°C, and washed three times with GST buffer. Native yeast whole-cell extract containing endogenous levels of Top3 and/or Rmi1 was prepared from a culture of KHSY2497 (MATα, *ura3Δ0*, *leu2Δ0*, *his3Δ1*, *lys2Δ0*, *TOP3.V5.VSV.KANMX6*, Open Biosystems), KHSY4695 (MATα, *ura3Δ0*, *leu2Δ0*, *his3Δ1*, *lys2Δ0*, *rmi1::HIS3*, *TOP3.V5.VSV.KANMX6*) or KHSY4696 (MATα, *ura3Δ0*, *leu2Δ0*, *his3Δ1*, *lys2Δ0*, *TOP3.V5.VSV.KANMX6*, *RMI1.myc.HIS3MX6*) grown at 30°C in YPD overnight. To construct a *top3Δ* yeast strain that expresses myc-epitope tagged Rmi1, a diploid generated by mating RDKY3837 (*MATa*, *ura3-52*, *trp1Δ63*, *his3Δ200*, *leu2Δ1*, *lys2Bgl*, *hom3-10*, *ade2Δ1*, *ade8*, *top3::TRP1*) and KHSY4696 (*MATα*, *ura3Δ0*, *leu2Δ0*, *his3Δ1*, *lys2Δ0, TOP3.V5.VSV.KANMX6*, *RMI1.myc.HIS3MX6*) was sporulated (38) to isolate a *top3::TRP1*, *RMI1.myc.HIS3MX6* haploid (KHSY4741) by genotyping on selective media. The presence of the *top3::TRP1* and *RMI1.myc.HIS3MX6* alleles was also confirmed by polymerase chain reaction. Yeast cells were collected by centrifugation at 2000 rpm for 4 min, washed and resuspended in Top3/Rmi1 buffer (50 mM Tris, pH 7.5, 0.01% NP-40, 5 mM β-glycerol phosphate, 2 mM magnesium acetate, 120 mM NaCl) plus HALT protease inhibitors (Pierce). The suspension was lysed via French press at 19 000 psi or in a BeadBeater (Biospec Products, Inc.) by beating three times for 1 min. Lysates were cleared by centrifugation at 14 000 rpm for 15 min at 4°C. Cleared yeast lysate of 20 (KHSY2497, KHSY4695) or 10 mg (KHSY4696, KHSY4741) was incubated with Sgs1-bound magnetic beads for 90 min at room temperature on a nutator. Beads were washed four times with Top3/Rmi1 buffer plus HALT protease inhibitors (Pierce) and boiled for 10 min in Laemmli buffer (BioRad). Beads were collected by centrifugation and eluted protein complexes were separated by 10% sodium dodecyl sulphate-polyacrylamide gel electrophoresis (SDS-PAGE). Presence of Sgs1 fragments, Top3 and Rmi1 and was determined by western blotting using monoclonal antibodies against GST (Covance), VSV (Sigma) and myc (Covance) epitopes, respectively.

### Gross-chromosomal rearrangement assay

Accumulation of cells that had undergone simultaneous inactivation of the *URA3* and *CAN1* genes on chromosome V was determined as previously described (39) except that cells were grown in the absence of leucine to select for the presence of the pRS415-derived plasmids expressing the desired *sgs1* mutants. Briefly, yeast strain KHSY1338 was transformed with derivatives of plasmid pRS415-SGS1 containing proline mutations (Supplementary Table SI) and grown to saturation at 30°C in 10 ml of SC-Leu. Cells were washed in water and plated on selective media containing canavanine (can) and 5-fluoro-orotic acid (5-FOA) to select for cells with inactive *CAN1* and *URA3* genes. Cells were also plated on SC-Leu media to obtain a viable cell count. After incubation at 30°C, viable cell count was determined after 3 days, and colonies on 5-FOA/can were counted after 5 days. Mutation rates and 95% confidence intervals were calculated from 6 to 16 cultures as previously described (39,40).

### Preparation of yeast whole-cell extracts by trichloroacetic acid extraction

To assess expression levels of Top3 and Rmi1 in *rmi1::HIS3* and *top3::TRP1* strains, respectively, yeast cultures were grown in YPD with vigorous shaking and 10 ODs were harvested by centrifugation at 2000 rpm for 2 min. To assess expression levels of sgs1-F30P and sgs1-H13P, the 3′-end of *SGS1* in pKHS481 was fused to the myc-epitope amplified from pFA6a-13Myc-HIS3MX6 (41) by gap repair of *Sac*I-linearized pKHS481 to generate pKHS596. *F30P* and *H13P* mutations were introduced into pKHS596 by QuikChange mutagenesis (Agilent Technologies) to generate pKHS598 and pKHS600, respectively. Cell pellets were washed in water and resuspended in ice-cold 20% trichloroacetic acid (TCA) and vortexed in a cell disruptor (USA Scientific) with acid-washed glass beads for 4 min at maximum speed. Cell lysate was cleared at 14 000 rpm for 3 min. The pellet was resuspended in Laemmli buffer, adjusted to neutral pH and boiled for 2 min before separation by 10% SDS-PAGE. Presence of Top3.VSV, Rmi1.myc, Sgs1.myc and GAPDH was determined by western blotting using monoclonal antibodies against VSV (Sigma) and myc (Covance) epitopes, and against GAPDH (Pierce), respectively.

## RESULTS

### The first 125 residues of the structurally disordered N-terminus of Sgs1 contain two transient α-helices

Sgs1 is a modular protein containing both ordered and disordered domains. The ATPase domain, zinc-binding domain, winged-helix domain and the HRDC domain make up the structurally ordered C-terminal half of Sgs1. In contrast, most of the N-terminal half of Sgs1 (residues 1–654) is predicted to be disordered (20,42). This is also the case for other members of the RecQ helicase family, most notably *S. pombe* Rqh1 and human BLM.

**Figure 1.**
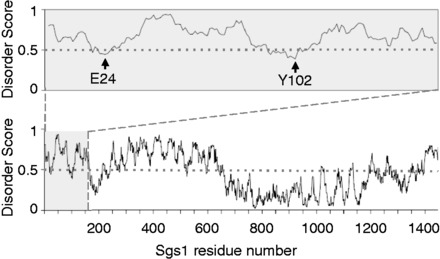
Prediction of intrinsically unstructured regions in Sgs1. Disorder scores are from IUPred (46) with scores >0.5 predicting disordered residues and scores <0.5 predicting ordered residues. Residues 1–158 (upper panel) are predicted to be mostly disordered with two short segments around residues E24 and Y102 dipping into the ordered region.

**Figure 2.**
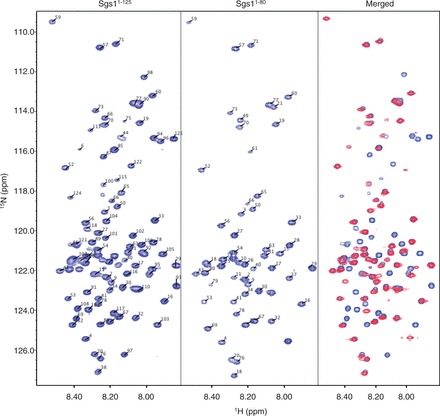
HSQC spectra of the first 125 residues of Sgs1 (Sgs1^1–125^) and the first 80 residues of Sgs1 (Sgs1^1–80^). Narrow chemical shift dispersion in the ^1^H dimension in both the HSQC spectra of the long (Sgs1^1–125^) and short (Sgs1^1–80^) peptide are consistent with a disordered peptide. The overlay of the long and short peptide (Merged) shows little discrepancy in the peak assignments between the two proteins, implying conservation of structural elements, even with the truncation.

**Figure 3.**
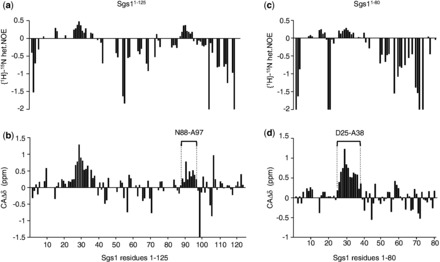
Measurement of NHNOE and secondary alpha carbon shifts (CAΔδ) of the Sgs1^1–125^ peptide and the Sgs1^1–80^ peptide. Consecutive positive values in the NHNOE plot for the Sgs1^1–125^ peptide (**a**) and the Sgs1^1–80^ peptide (**c**) indicate regions with a slower rotational correlation time that may adopt secondary structure. Consecutive positive secondary alpha carbon chemical shifts (CAΔδ) between residues 88 and 97 (**b**) and between residues 25 and 38 (**d**) indicate the presence of α-helical secondary structure in the unbound Sgs1 peptide as compared with standard chemical shifts in a random coil library (48,50).

A previous study has shown that the first 158 residues of Sgs1 are sufficient for binding to the topoisomerase Top3 (18). It is well established that short segments within longer disordered regions will undergo coupled folding and binding in the presence of protein binding partners (43–45). Disorder predictors like IUPred (46) will frequently display short dips into the ordered region (disorder tendency < 0.5) that correspond to these protein binding sites, and it is expected that these regions will contain some degree of transient secondary structure. The lowest dips in the IUPred plot of the first 158 residues of Sgs1 correspond to residues E24 and Y102 (Figure 1). To determine whether these small segments within the disordered N-terminus of Sgs1 could adopt functionally significant secondary structures, we characterized the solution structure of the first 125 residues of Sgs1 using NMR spectroscopy. Single (^15^N)- and double (^15^N/^13^C)-labeled samples of Sgs1^1^^−125^ were overexpressed in *E. coli* and purified to apparent homogeneity. The double-labeled sample was used to measure the HSQC spectrum (Figure 2) as well as the triple resonance spectra that were used to make resonance assignments. The HSQC spectrum shows narrow chemical shift dispersion in the ^1^H dimension (7.85–8.5 ppm), consistent with a disordered peptide (47–49). The ^15^N-labeled sample was used to measure the NHNOE. NHNOE values are sensitive to the rotational correlation time for the residue of interest. In disordered regions, small positive NHNOE values indicate regions that are less dynamic and typically correlate with the presence of transient secondary structure, and negative NHNOE values indicate highly dynamic regions. The NHNOE values observed for Sgs1^1^^−125^ are consistent with a mostly disordered protein that contains two transiently ordered regions centered on residues F30 and E92 (Figure 3a). Alpha carbon secondary chemical shifts (CAΔδ) were calculated for every residue by subtracting the amino acid–specific random coil chemical shift values for CA from the measured values (50). This is a reliable method for identifying the presence of transient secondary structure in IDPs (51–53). The presence of transient α-helical secondary structure in Sgs1^1^^−125^ was indicated by consecutive positive CAΔδ values for residues 23–34 and 88–97 (Figure 3b).

Several clusters of overlapping resonances in the HSQC and HNCACB spectra, and repeating amino acid motifs (e.g. Thr-Ala-Thr) limited resonance assignments to 77% of the nonproline residues for the Sgs1^1^^−125^ fragment. Several of the residues that could not be assigned were in or near the two transient α-helical segments preventing an identification of the helix boundaries. To develop a more complete picture of the first helical region, NMR analysis of a shorter Sgs1 fragment containing residues 1–80 (Sgs1^1^^−80^) was performed. Using this fragment, we were able to assign 93% of the nonproline resonances in the HSQC spectrum (Figure 2) and to fill in the gaps in the secondary ^13^C_α_ chemical shift analysis (Figure 3c and d). The overlap between the HSQC spectra of the Sgs1^1^^−80^ and the Sgs1^1^^−125^ peptides indicates that elimination of 36% of the residues of the Sgs1^1^^−125^ peptide (45 residues) did not affect the solution structure of the first 80 residues of Sgs1, consistent with this being a disordered region. Secondary ^13^C_α_ chemical shift analysis indicates the presence of α-helical secondary structure for residues 25–38 and residues 88–97 within this disordered region (Figure 3b and d). However, as mentioned above, helical states for both regions are transient because secondary shift values of > 2.6 δppm would be expected for ^13^C_α_ in a persistent α-helix (51).

### Functional mapping of α-helices by proline mutagenesis

**Figure 4.**
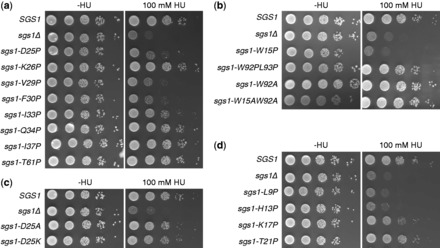
HU hypersensitivity of cells expressing *sgs1* alleles with mutations in (**a**) the α-helical region spanning residues 25–38 and (**b**) the α-helical region spanning residues 88–97. The wild-type phenotype exhibited by cells expressing W15A and W92A mutants of Sgs1 also demonstrates that these aromatic residues are not involved in stacking. T61P was included as a control for a disordered residue. (**c**) Replacing the N-cap residue D25 with basic (D25K) or neutral (D25A) residues that are poor N-caps, but have strong α-helical propensity, does not affect Sgs1 function. (**d**) Extending proline mutagenesis N-terminally of the first α-helical region reveals additional functional residues (L9, H13, H17).

To determine if the transient α-helical structures for residues 25–38 and 88–97 are important for Sgs1 function, residues with the highest NHNOE and CAΔδ values in each helical region were replaced with prolines—a known helix breaker. V29 and F30 in the first helical region and W92 and L93 in the second helical region were changed to proline in the context of full-length Sgs1. Cells expressing the mutant helicases were plated on media containing 100 mM of the DNA-damaging agent HU (Figure 4). While the *sgs1-V29P* and *sgs1-F30P* mutants were as sensitive to HU as the *sgs1Δ* mutant, neither the W92P nor the L93P mutation caused increased sensitivity (Figure 4a and b), indicating that the α-helical structure centered on V29 and F30 contributes to Sgs1’s role in DNA damage repair, whereas that centered on W92 and L93 does not.

According to the NHNOE and CAΔδ values, the strongest helical region in the first 125 residues of Sgs1 extends from residues 25 to 38. To determine the functional distance that this helical region extends on both sides of V29 and F30, residues were mutated according to the expected *i*, *i* + 4 intramolecular hydrogen-bonding pattern of a typical α-helix. The mutants using V29 as a starting point, therefore, were D25P, I33P and I37P and those based on F30 were K26P and Q34P. Because proline substitutions of disordered residues near the ordered region would not be predicted to affect Sgs1 function, a T61P mutation (IUPred disorder score: 0.73) was included as a negative control. The *sgs1-K26P*, *sgs1-Q34P*, *sgs1-I37P* and *sgs1-T61P* mutants exhibited wild-type levels of HU sensitivity, whereas the *sgs1-D25P* and *sgs1-I33P* mutants were hypersensitive, with a gradual decrease in functional impairment of Sgs1 being observed between proline substitutions near the N-terminus of the helix and those near the C-terminus (Figure 4a). These observations are consistent with the functional α-helix extending from residues 25 to 33.

**Figure 5.**
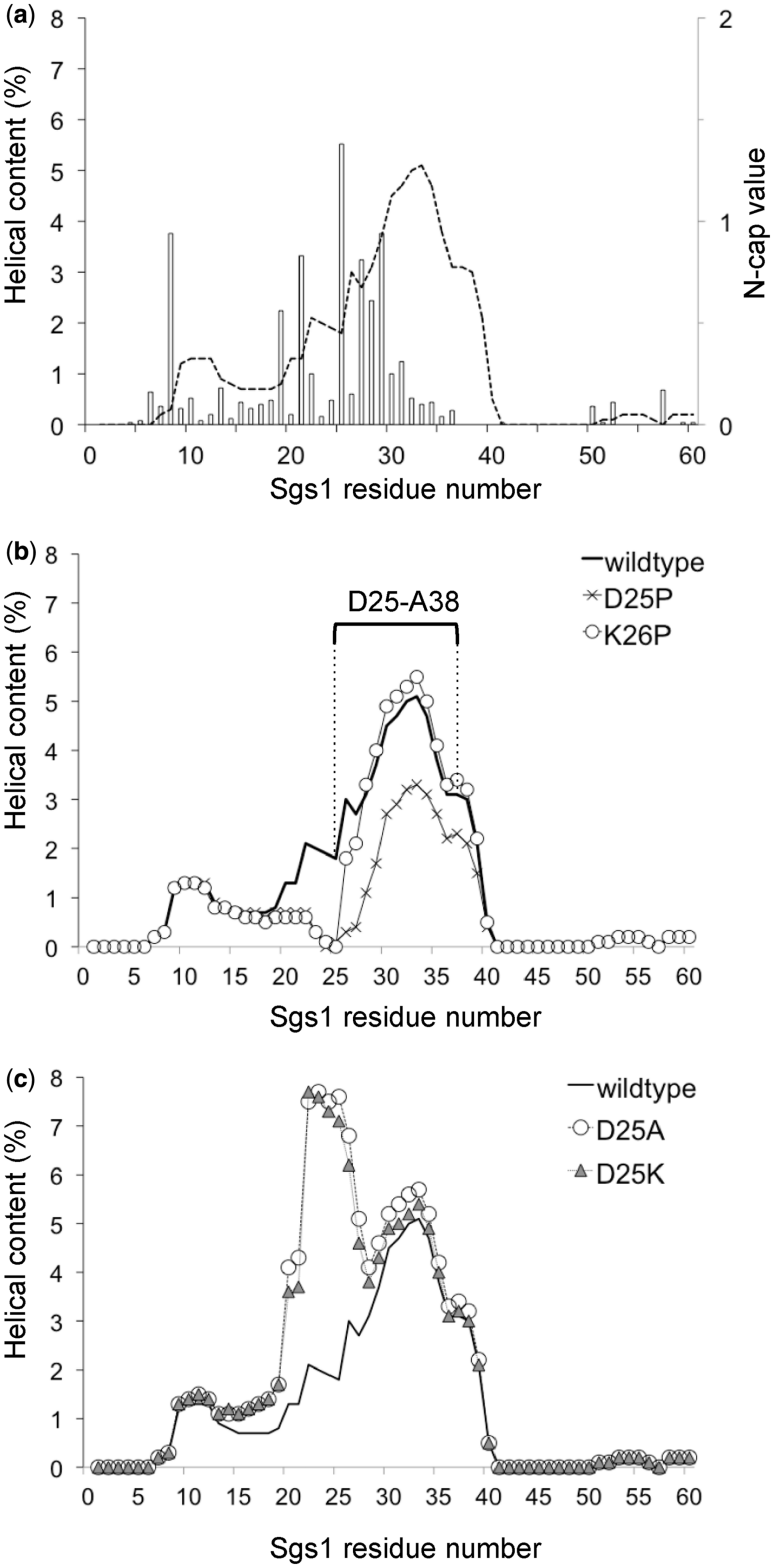
AGADIR (57) prediction of the helical content of the N-terminus of Sgs1. (**a**) In wild-type Sgs1 (dotted line), a prominent peak of helical propensity is predicted at residue I33 and a smaller peak at residues R10–E12. Residue D25 received the highest N-cap score (open columns). (**b**) The deleterious D25P mutation is predicted to reduce the helical content of the D25–A38 region, whereas the nondeleterious K26P mutation is not. (**c**) Replacing the N-cap residue D25 with residues that have excellent helical propensity, but are poor N-cap residues (lysine, alanine), is not predicted to reduce helical content of the D25–A38 region, but predicts an N-terminal extension of the helical region with a new peak of helical content at residue Q23 in the D25A mutant and at residue L22 in the D25K mutant.

Whereas the lack of an effect of proline in position 26 argues against K26 being an internal residue of α-helix, our findings are consistent with K26 being in the first helical turn, more specifically in the N1 position, where proline is tolerated (54,55), whereas D25—as the N-cap residue (56)—defines the N-terminal helix boundary. Indeed, the AGADIR algorithm (57) identified a prominent peak of helical propensity centering on residue I33, and D25 received the highest N-cap score (Figure 5a). Consistent with the results of the DNA-damage-sensitivity assay, AGADIR predicted reduced helical content for the D25P mutant, but not for the K26P mutant (Figure 5b). Removing the N-cap by replacing the aspartic acid residue at position 25 with basic (D25K) or neutral (D25A) residues, which have excellent helical propensity, but are poor N-cap residues (58), leads to N-terminal extension of the helix in AGADIR (Figure 5c). This increase in helical content in the *sgs1-D25K* and *sgs1-D25A* mutants did not impair Sgs1 function *in vivo* (Figure 4c).

Further extending the proline mutagenesis starting from V29 toward the N-terminus revealed wild-type levels of HU sensitivity for *sgs1-T21P*, consistent with D25 defining the N-terminal end of the α-helix. In contrast, the *sgs1-K17P*, *sgs1-W15P*, *sgs1-H13P* and *sgs1-L9P* mutants were more sensitive to HU than cells expressing wild-type Sgs1, indicating that this region is also critical for Sgs1 function (Figure 4b and d). The stretch of consecutive positive CAΔδ values for N8 to R11 is consistent with α-helical propensity and the HU hypersensitivity assay suggests that it extends C-terminally to residue H17. At first sight, the negative CAΔδ value for W15 seems to indicate that W15 is not in a transient helical structure (Figure 3d). If this is the case then it suggests that any helical structure in the bound state is not contiguous from residue 8 to 17. However, the inconsistent CAΔδ value for W15 could be owing to the inaccuracies associated with the random coil chemical shift library used for calculating the secondary chemical shifts (50) or related to an anomalous effect on the CA shift that results from the partial charge of the H13 and H17 side chains. Consistent with W15 being an α-helix, substituting the tryptophan with other residues with good helical propensity, such as alanine or arginine, did not affect Sgs1 function in the DNA-damage hypersensitivity assay (W15A, Figure 4b) or its ability to induce slow growth in the *sgs1Δ top3Δ* strain (59). However, α-helical content in this region could not be further assessed as assignments, and therefore NHNOE and CAΔδ values for residues S6, E12, H13 and K14 were not available owing to overlapping resonances in the HSQC spectra of both Sgs1^1^^−80^ and Sgs1^1^^−125^. That W15 and W92 could be changed to nonaromatic residues without increasing sensitivity of cells to DNA damaging agents (Figure 4b) also shows that these two residues are not involved in stacking interactions with each other, with other aromatic residues in the region, or with DNA (60,61), or at least that such stacking interactions are not important for the role of Sgs1 in suppressing HU hypersensitivity.

**Figure 6.**
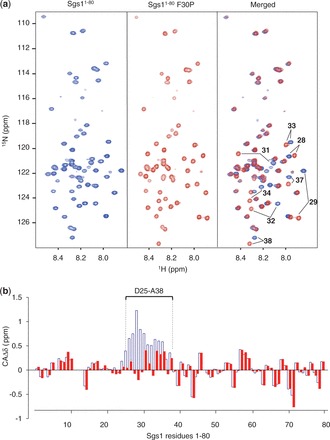
HSQC spectra and secondary chemical shift (CAΔδ) analysis of the first 80 residues of Sgs1 with a proline substitution at residue 30 (sgs1^1–80^-F30P). (**a**) The overlay (Merged) of the HSQC spectra of wild-type Sgs1 (blue) and the sgs1-F30P mutant (red) reveals shifts in the peak assignments for residues F28, V29, Q31, A32, I33, Q34, I37 and A38, which form a transient α-helix in wild-type Sgs1. (**b**) Consecutive positive secondary alpha carbon chemical shifts (CAΔδ) between residues D25–A38 in wild-type Sgs1 (open blue columns), which indicate the presence of α-helical secondary structure in the unbound Sgs1 peptide, are markedly reduced in the Sgs1-F30P mutant (red filled columns).

To verify that proline mutations that cause HU hypersensitivity indeed disrupt the α-helix between residues D25 and A38, we analyzed the solution structure of the sgs1^1^^−80^-F30P mutant by NMR (Figure 6). We found that the resonances that shifted notably in the HSQC spectrum of the F30P mutant compared with the wild type were limited to residues F28–A38 (Figure 6a, Merged), suggesting that changes induced by the F30P mutation are probably localized to the α-helix. Indeed, the consecutive positive secondary alpha carbon chemical shifts (CAΔδ) between residues D25 and A38 in wild-type Sgs1, which indicate the presence of α-helical secondary structure, were markedly reduced in the F30P mutant (Figure 6b), demonstrating that a proline at position 30 is sufficient to prevent the formation of the α-helix between residues 25 and 38. We also confirmed that proline mutations that disrupt α-helical content in the N8–H17 region or the D25–A38 region and cause the highest HU sensitivity (H13P, F30P) do not affect Sgs1 expression levels and stability (Supplementary Figure S1).

### Disruption of transient α-helices impairs complex formation between Sgs1, Top3 and Rmi1

**Figure 7.**
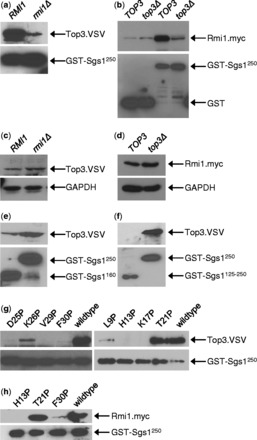
Loss of function of Sgs1 proline mutants is due to loss of Top3 and Rmi1 binding. Sgs1 proline mutants were expressed as N-terminal GST fusions in *E. coli* and purified by binding to glutathione beads. Top3 and Rmi1 were obtained from native whole-cell extracts of yeast strains KHSY2497 (*RMI1*), KHSY4695 (*rmi1Δ*) or KHSY4696 (*RMI1.MYC*), KHSY4741 (*top3Δ)*, which express epitope-tagged Top3 and/or Rmi1 from their chromosomal loci under their native promoters. (**a**) Binding of Top3 to the Sgs1^1–250^ peptide is Rmi1-dependent. (**b**) Binding of Rmi1 to the Sgs1^1–250^ peptide is Top3-dependent. (**c**) Deletion of *RMI1* does not lead to loss of Top3 expression. (**d**) Deletion of *TOP3* does not lead to loss of Rmi1 expression. (**e**) The Sgs1^1–250^ peptide binds Top3 more strongly than the shorter Sgs1^1–160^ peptide. (**f**) The Sgs1^125–250^ peptide does not bind Top3, indicating that critical residues for Top3 binding are located in the first 125 residues of Sgs1. (**g**) Proline mutations at L9, H13, K17, D25, V29 and F30, but not at T21 and K26, reduce binding of Sgs1^1–250^ to Top3. (**h**) Proline mutations at H13 and F30, which reduce binding of Sgs1^1–250^ to Top3, also reduce binding to Rmi1.

The disordered region of Sgs1 where the transient α-helices were identified binds to the Type-1A topoisomerase Top3 (18). To test if HU hypersensitivity caused by proline mutations in this region is owing to the disruption of transient helices that are required for the interaction between Sgs1 and Top3, the ability of various sgs1 mutants to form a complex with Top3 was assessed *in vitro*. Because overexpression of full-length Sgs1 leads to insolubility (62,63), we chose the N-terminal 250 residues of Sgs1 and expressed them as an N-terminal GST fusion in *E. coli*. This Sgs1^1^^−250^ fragment pulled down endogenous Top3 from native yeast whole-cell extract in an Rmi1-dependent manner (Figure 7a). Similarly, binding of Rmi1 to Sgs1^1^^−^^250^ was reduced in the absence of Top3 (Figure 7b), suggesting that Top3 and Rmi1 depend on each other for binding to the N-terminal 250 residues. Despite the effect on Sgs1 binding, expression levels of Top3 and Rmi1 were not affected by the absence of Rmi1 and Top3, respectively (Figure 7c and d). Sgs1^1^^−250^ binds to Top3 more strongly than Sgs1^1^^−160^ and, similar to what has been reported previously for an Sgs1 fragment comprising residues 107–283 (19), Sgs1^125^^−250^ did not bind to Top3 (Figure 7e and f). When we introduced L9P, H13P, K17P, D25P, V29P and F30P mutations into the Sgs1^1^^−250^ fragment, its ability to pull down Top3 from cell extracts was diminished, whereas the T21P and K26P mutants were still able to bind Top3 (Figure 7g). Mutations of Sgs1 that disrupted binding to Top3 also disrupted binding to Rmi1 (Figure 7h).

### Integrity of transient α-helices is critical for maintaining chromosomal stability

Lack of Sgs1 or disruption of its conserved C-terminal helicase core domain leads to mitotic hyperrecombination and a moderate increase in the accumulation of gross-chromosomal rearrangements (GCRs), including translocations between nonallelic sites (39,64,65). To determine if the inability of Sgs1 to interact with Top3 and Rmi1 also leads to increased genome instability, we tested the ability of D25P, K26P, V29P, F30P and I33P mutants of full-length Sgs1 expressed from a CEN/ARS plasmid to suppress the elevated GCR rate of an *sgs1Δ* mutant. Mirroring the results of the HU hypersensitivity assay, D25P, V29P, F30P and I33P were unable to complement the defects of *sgs1Δ* cells, whereas cells expressing the K26P mutant accumulated GCRs at a similar rate as cells expressing wild-type Sgs1 (Table 1).

**Table 1.** Effect of proline substitutions in the transient α-helix between residues D25 and A38 of Sgs1 on the rate of accumulating GCRs

Relevant genotype	Plasmid	GCR rate (Can^r^ 5–FOA^r ^× 10^−8^)	95% CI^a^ (Can^r^ 5-FOA^r ^× 10^−8^)	Increase over wild type (*SGS1*)	
*SGS1*	pKHS481	58	34–73	1	
*sgs1-D25P*	pKHS494	334	260–789	6
*sgs1-K26P*	pKHS500	71	39–132	1
*sgs1-V29P*	pKHS492	320	189–352	6
*sgs1-F30P*	pKHS482	704	194–996	12
*sgs1-I33P*	pKHS496	211	165–255	4

^a^95% confidence intervals were calculated according to Nair (40), with nonoverlapping confidence intervals indicating statistically significant differences (α < 0.05) between median GCR rates.

## DISCUSSION

In the prokaryote-to-eukaryote transition, some members of the RecQ helicase family acquired long N-terminal regions that precede the ATPase domain of the helicase core. In Sgs1, the only RecQ homolog in *S. cerevisiae*, this N-terminal region is ∼650 amino acids long, making up ∼45% of the 1447-residue long protein. This entire region is predicted to be intrinsically disordered and to contain several short segments of transient secondary structure (Figure 1). Using NMR spectroscopy, we have demonstrated that the first 125 residues of this N-terminal region of Sgs1 are intrinsically disordered in the unbound solution state with two short segments, between residues 25–38 and 88–97, that adopt transient α-helical structure. Transient α-helices in disordered regions of proteins are often stabilized by interactions with a binding partner (43–45,66,67). This principle was used to rationally design single residue substitutions that disrupted the transient α-helical structures of residues 25–38 and 88–97, and the effects of these mutations on Sgs1 function were tested *in vitro* and *in vivo*. Substitution of residues D25, V29, F30 and I33 with the α-helix breaker proline impaired Sgs1 function *in vivo*, as evidenced by increased sensitivity to DNA damage and increased chromosome instability, and reduced binding of Top3 and Rmi1 to Sgs1 *in vitro*. Additional proline mutagenesis following the *i*, *i* − *4* α-helix pattern revealed that L9, H13 and K17 were critical for the same Sgs1 functions as the D25–A38 α-helix.

Our work demonstrates that the integrity of a transient α-helix is required for the *in vivo* function of Sgs1 and the binding of Sgs1 to Top3 and Rmi1. This helps explain why previous attempts to identify functionally critical single residues through alanine scanning of the region were unsuccessful (K4A, P5A, L9A) (68). Alanine scanning is often useful for identifying residues important for catalytic function, such as the ATPase activity of Sgs1 (K706A in the Walker A motif). However, the effectiveness of this approach to detect functionally important structural motifs in disordered segments, such as transient α-helices, is hampered by the high helical propensity of alanine and will depend on whether the substitution occurs at a residue that forms part of the binding interface (58). Substitution with lysine and valine residues, which also have excellent helical propensity, also had no effect on Sgs1 function (D25K, Figure 4c; D25V (59), whereas a proline substitution at this same residue disrupted function (D25P, Figure 4a). Even amino acid residues that have lower helical propensity and are therefore not commonly found in α-helices, such as glycine and serine, are not necessarily successful at disrupting transient α-helices when introduced as single-residue substitutions. For example, the E12G and H13S mutations by themselves were insufficient to disrupt the interaction between Sgs1 and Top3, but were effective when combined (68). Rationally designing mutations based on residue-specific knowledge of transient secondary structure provided a direct test of structure/activity relationships for Sgs1 (and presumably other IDPs) that could only be realized by combining a high-resolution structural approach, like NMR, with the *in vivo* and *in vitro* functional tests that can be performed in a model organism like *S. cerevisiae*. While this type of multiscale approach has commonly been used to interrogate structure/activity relationships for ordered proteins, the widespread application of this approach to IDPs/IDRs has been hampered by a lack of understanding of the general rules that connect their dynamic structures to their function. We believe our study helps clarify an approach that can be consistently applied to identify the functionally critical regions of IDPs/IDRs.

What functional advantages might the long, intrinsically disordered N-terminal tail provide to Sgs1? One possibility is that it contains multiple protein interaction sites, in addition to Top3/Rmi1. This hypothesis is supported by multiple dips below the 0.5 threshold in the IUPred plot (Figure 1) and the fact that Sgs1 binds Top2, Rad16, Rpa70, Dna2 and Mre11 at sites that map to the disordered N-terminus, although the discrete binding sites have not been identified (6–8,13). Sgs1 may need to bind several of these proteins, sequentially or concurrently, in the same process. For example, the Sgs1/Top3/Rmi1 complex is instrumental in DNA resection during double-strand break (DSB) repair in a reaction analogous to that performed by the RecBCD complex in bacteria. In this model, which was recently proposed by Cejka *et al.* (13), the Sgs1/Top3/Rmi1 complex is first recruited to the DSB by physically interacting with the Mre11 subunit of the Mre11/Rad50/Xrs2 complex. Subsequently, the Sgs1/Top3/Rmi1 complex physically interacts with Dna2 to stimulate preferential degradation of the 5′-end and with replication protein A (RPA) to protect the 3′-end. Still other physical interactions at the N-terminal tail, including those with Rad16 and Top2, are likely to be important for roles of the Sgs1/Top3/Rmi1 complex in DNA repair and chromosome segregation. Conformational flexibility may also be crucial to accommodating the various structures and sizes of DNA substrates that the Sgs1/Top3/Rmi1 complex acts on, which range from simple double-stranded or splayed ends to hairpins, quadruplexes, Holliday junctions and telomeres.

In *E. coli*, RecQ and Top3 interact functionally, but not physically. One advantage of gaining physical contact between Sgs1 and Top3 would be the ability of one subunit in the complex to regulate another subunit’s enzymatic activity. Tight coordination between a Type-IA topoisomerase activity, such as exhibited by Top3, and DNA-dependent ATPase activity, such as exhibited by the helicase core of Sgs1, can be seen in the reverse gyrases of thermophile and hyperthermophile bacteria and archaea, where the two activities are either contained in a single polypeptide (69,70) or are encoded by two separate genes (71). In these enzymes, the topoisomerase domain has been found to reduce the activity of the helicase-like ATPase domain (72) and, conversely, the ATPase domain has been shown to inhibit the supercoil relaxation activity of the topoisomerase subunit to induce positive supercoiling (71). Inhibition of the helicase activity of the human Werner syndrome helicase WRN by its associated Type-1B topoisomerase Topo I hints at the possibility of coordination between the two activities also in RecQ-like helicases. Similarly, in Sgs1, deletion of the Top3 contact site (*sgs1Δ1-158*) causes a more severe phenotype than that caused by the absence of Sgs1 (3), which could be explained by Top3 binding having an inhibitory effect on the ATPase activity of Sgs1.

**Figure 8.**
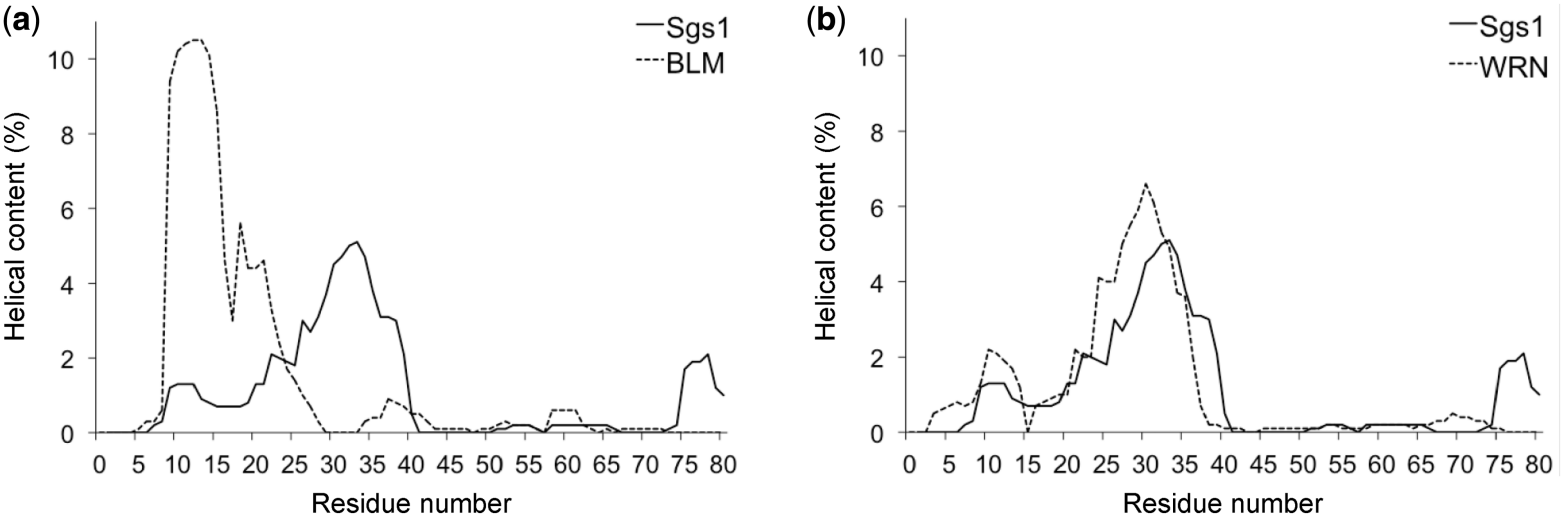
Helical content prediction for the N-termini of Sgs1, WRN and BLM by AGADIR (57). (**a**) In human BLM, which binds to the human Top3 homologue Topo IIIα, a prominent peak of helical content is predicted at residues Q12 and L13, which corresponds to the small R10–E12 peak in Sgs1. A peak corresponding to that at residue I33 in Sgs1 is not predicted in BLM, in part because of a proline residue at position 30. (**b**) The distribution of predicted helical content for the N-terminus of human WRN, which binds to Topo I, but has not been shown to bind to Topo IIIα, is similar to Sgs1, with two prominent peaks at residues E10 and A30, corresponding to similar peaks at R10–E12 and I33 in Sgs1.

The interaction with a Type-1 topoisomerase has been preserved in at least four of the five human RecQ-like helicases: BLM, WRN, RecQL1 and the long isoform of RecQL5. Like Sgs1, BLM and WRN interact with Topo IIIα (Type IA) and Topo I (Type IB), respectively, at the far end of a long N-terminal tail (12). Human RecQL1 was also found to interact with Topo IIIα, whereas the long isoform of RecQL5 (RecQL5β) co-immunoprecipitated with Topo IIIα and Topo IIIβ (10,11). The predicted helical content of the N-terminus of BLM does not resemble that of the Top3/Rmi1 contact site between residues 25 and 38 in Sgs1, which appears to be the result of a proline substitution in BLM at position 30 (Figure 8a). Instead, the helical content in the segment starting with residue L9, which is weak in Sgs1, is predicted to be dominant in BLM. Thus, although both BLM and Sgs1 interact with topoisomerase 3 at the N-terminus, the structural elements in the two proteins that mediate this interaction may not be conserved. This is also supported by the finding that the C-terminal 156 residues of BLM also bind to Topo IIIα (12), whereas only the N-terminus of Sgs1 interacts with Top3. Strikingly, the predicted helical content for residues N23 to R36 in WRN is nearly a perfect match to that of the confirmed α-helix in Sgs1 (Figure 8b). However, WRN has not been shown to interact with Topo IIIα (10), possibly owing to the insertion of the exonuclease domain just downstream of this site, and it will be interesting to test if residues N23–R36 of WRN can provide a contact site for the Top3/Rmi1 complex when placed in Sgs1. In the case of *S. pombe* Rqh1, the first 322 N-terminal residues are required for interaction with Top3 (9). Although helical content is not predicted for the first 100 residues of this region, noticeable helical content is evident for the 27-residue region between residues H264 and R291 and the 15-residue region between residues D112 and Q127, which could be investigated as putative Top3 binding sites (Supplementary Figure S2). Although Topo IIIα also binds full-length RecQL1 and RecQL5 (10,11), the binding regions in these two human RecQ homologs have not yet been narrowed down.

Applying the same NMR-based structure–function analysis to the remaining 525 residues of the disordered N-terminal tail of Sgs1 (and the tails of the other long RecQ-like helicases) will help to identify additional structural elements, either transient or persistent, that serve as molecular recognition elements for protein partners or DNA, and allow for the rational design of new separation of function alleles that encode mutants of RecQ-like helicases with single residue substitutions that are defective in discrete cellular functions.

## SUPPLEMENTARY DATA

Supplementary Data are available at NAR Online.

## FUNDING

National Institutes of Health [R01GM081425 to K.H.S.]; American Cancer Society [RSG0728901GMC to G.W.D.]; National Science Foundation [MCB0939014 to G.W.D.]. Funding for open access charge: NIH [R01GM081425 to K.H.S.].

*Conflict of interest statement*. None declared.

## Supplementary Material

Supplementary Data
