# Supplementary material for: A transient α-helical molecular recognition element in the disordered N-terminus of the Sgs1 helicase is critical for chromosome stability and binding of Top3/Rmi1
Source: Nucleic Acids Res. 2013 Sep 14;41(22):10215–27. doi: 10.1093/nar/gkt817 (PMC3905885; doi:10.1093/nar/gkt817)
Supplement: Supplementary Data [file supp_41_22_10215__index.html]

A transient α-helical molecular recognition element in the disordered N-terminus of the Sgs1 helicase is critical for chromosome stability and binding of Top3/Rmi1 — A transient α-helical molecular recognition element in the disordered N-terminus of the Sgs1 helicase is critical for chromosome stability and binding of Top3/Rmi1 — Supplementary Data 

# A transient α-helical molecular recognition element in the disordered N-terminus of the Sgs1 helicase is critical for chromosome stability and binding of Top3/Rmi1

## Supplementary Data

files

**Files in this Data Supplement:**

- Supplementary Data - pdf file
