# Supplementary material for: A transient α-helical molecular recognition element in the disordered N-terminus of the Sgs1 helicase is critical for chromosome stability and binding of Top3/Rmi1
Source: Nucleic Acids Res. 2013 Sep 14;41(22):10215–27. doi: 10.1093/nar/gkt817 (PMC3905885; doi:10.1093/nar/gkt817)
Supplement: Supplementary Data [file supp_gkt817_nar-01124-m-2013-File010.pdf]

Supplementary Table 1. Plasmids used in this study

Plasmid	Description
pRS415	*CEN/ARS, LEU2*
pKHS443	*pET28a-SGS11-N1-125*
pKHS460	*pGEX-6p-2-SGS1-N125-250*
pKHS462	*pET28a-SGS1-N1-250*
pKHS463	*pET28a-SGS1-N1-80*
pKHS466	*pGEX-6p-2-SGS1-N1-160*
pKHS481	*pRS415-SGS1*[i]
pKHS482	*pRS415-SGS1-F30P*
pKHS484	*pRS415-SGS1-W92PL93P*
pKHS485	*pRS415-SGS1-L93P*
pKHS489	*pRS415 -SGS1-K26P*
pKHS492	*pRS415-SGS1-V29P*
pKHS494	*pRS415-SGS1-D25P*
pKHS496	*pRS415-SGS1-I33P*
pKHS497	*pRS415-SGS1-Q34P*
pKHS546	*pGEX-6p-2-SGS1-N1-250-K26P*
pKHS547	*pGEX-6p-2-SGS1-N1-250-V29P*
pKHS548	*pGEX-6p-SGS1-N1-250-F30P*
pKHS582	*pRS415-SGS1-L9P*
pKHS583	*pRS415-SGS1-H13P*
pKHS584	*pGEX-6p-2-SGS1-N1-250-L9P*
pKHS585	*pGEX-6p-2-SGS-N1-250-H13P*
pKHS586	*pGEX-6p-2-SGS1-N1-250-T21P*
pKHS587	*pGEX-6p-2-SGS-1N1-250-K17P*
pKHS588	*pRS415-SGS1-L181P*
pKHS589	*pRS415-SGS1-L215P*
pKHS590	*pRS415-SGS1-T61P*
pKHS591	*pRS415-SGS1-L176P*
pKHS592	*pRS415-SGS1-W15P*
pKHS594	*pRS415-SGS1-I37P*
pKHS595	*pGEX-6p-2-SGS1-N1-250-D25P*
pKHS596	*pRS415-SGS1.MYC.HIS3MX6*
pKHS598	*pRS415-SGS1-F30P.MYC.HIS3MX6*
pKHS600	*pRS415-SGS1-H13P.MYC.HIS3MX6*
pKHS602	*pET28a-SGS1-N1-80-F30P*

[i] Gift from Dr. Steven Brill (Rutgers University)

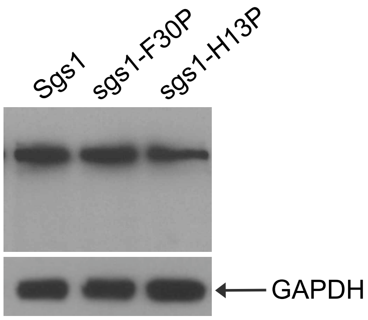

Figure S1. Proline substitutions in Sgs1 that cause hypersensitivity to the DNA-damaging agent hydroxyurea do not affect expression levels of the sgs1 mutant proteins. The *sgs1Δ* mutant KHSY1338 was transformed with plasmids pKHS596 (Sgs1.myc), pKHS598 (sgs1-F30P.myc) or pKHS600 (sgs1-H13P.myc). Whole cell extracts were prepared by TCA extraction from mid-log phase cultures and separated by 10% SDS-PAGE. Wildtype Sgs1 and the sgs1-F30P, sgs1-H13P mutants were detected by Western blotting with monoclonal antibody against the C-terminal myc-epitope. GAPDH was detected by a monoclonal antibody against GAPDH.

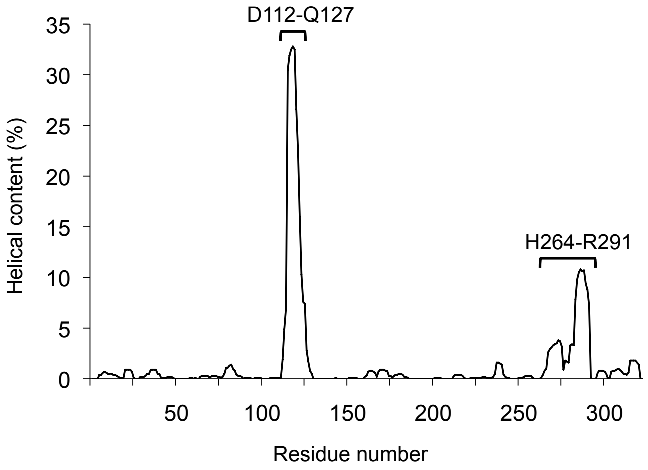

Figure S2. Helical content prediction for the N-terminus of *S. pombe* Rqh1 by AGADIR (57). The distribution of helical content for the first 322 residues, which are required for interaction with Top3 (9), are shown. The first prominent peak is predicted at residue M117 within a 15-residue segment spanning from D112 to Q127, and a second peak at I286 within a 27-residue segment spanning from H264 to R291.
